# Cu_γ_ (γ = 1–3)-Modified MoS_2_ Monolayer as a Gas Sensor for Detecting C_4_F_7_N and Its Decomposition Components

**DOI:** 10.3390/nano12162829

**Published:** 2022-08-17

**Authors:** Changyun Li, Peigang Chen, Yongjin Yu, Chuanyang Li

**Affiliations:** 1 College of Electrical Engineering and Automation, Shandong University of Science and Technology, Qingdao 266590, China; 2 Department of Electrical Engineering, Tsinghua University, Beijing 100084, China

**Keywords:** first principle, C_4_F_7_N decomposition components, gas sensors, adsorption, applied electric field

## Abstract

Perfluorinated isobutyronitrile (C_4_F_7_N) is favored in electrical engineering because it is an environmentally friendly gas-insulating medium with a low greenhouse effect. Unfortunately, under the influence of electricity and over-heating, its decomposition results in the deterioration of its insulating properties, which potentially leads to partial discharge or even gas breakdown. In this paper, the adsorption behavior of C_4_F_7_N gas and its toxic decomposition product, acetonitrile (C_2_N_2_), on MoS_2_ surfaces doped with small copper clusters was investigated by calculating the adsorption energy and density of states, etc. The effects of multiple initial adsorption positions as well as externally applied electric fields were also taken into account. The results depict that the maximum adsorption energy of C_4_F_7_N on the Cu_γ_ (γ = 1–3)-MoS_2_ surface gradually decreases with the increase in *γ*. The Cu_3_-modified MoS_2_ is most suitable for use as a resistive-based gas-sensitive sensor substrate. This paper provides the theoretical foundation for the maintenance of future power equipment with environmentally friendly insulating gas.

## 1. Introduction

Sulfur hexafluoride (SF_6_) gas, due to its excellent insulating properties such as its high dielectric strength and arc quenching performance, is mostly and widely used in Gas-insulated Switchgear (GIS), Gas-insulated Line (GIL), Gas-insulated Cabinet (GIC) and other gas-insulated power equipment in electric power transmission and distribution projects. However, SF_6_ is a powerful greenhouse gas with about 23,500 times the global warming potential (GWP) of that of CO_2_ [1]. The green development of global electrical engineering is an inevitable requirement in sake of environmental protection, which required that the use of SF_6_ must be reduced. To meet both the high dielectric strength and environmental protection demands, C_4_F_7_N, with a GWP of 2090 and a dielectric strength two times higher than that of SF_6_, was found by the 3M company in 2019. The first GIC using C_4_F_7_N gas was developed by the China Institute of Electrical Technology in the same year.

However, in the operating of electrical equipment, C_4_F_7_N is subjected to electrical and thermal stresses, resulting in the degradation of the insulating gas. By monitoring the composition and content of the pyrolysis product, the insulating property of C_4_F_7_N can be characterized. Hence, to ensure the safe and stable operation of C_4_F_7_N gas-insulated electric power equipment, it is necessary to detect the decomposition products of C_4_F_7_N in real time so as to diagnose early insulation faults. To achieve this goal, it is necessary to clarify the byproduct components of C_4_F_7_N and master the fundamental detection principle of each component.

G.Q. Zhang et al. investigated the partial discharge (PD) decomposition characteristics of C_4_F_7_N mixed with CO_2_ with a pin-plate discharge test model, and the results showed that the pyrolysis components of C_4_F_7_N mainly contained C_2_F_6_, C_2_F_4_, C_3_F_6_, CF_3_CN and C_2_N_2_ [2]. X. Zhang et al. conducted a 96 h PD decomposition test on C_4_F_7_N/CO_2_ gas mixtures using a needle-plate electrode and clarified that the characteristic decomposition components include CO, CF_4_, C_2_F_6_, CF_3_CN, C_2_N_2_ and COF_2_ [3]. Further, they showed that the contents of CO, CF_4_, C_2_F_6_, CF_3_CN and C_2_N_2_ were significantly higher than those of other components. In 2017, B. Radisavljevic et al. investigated the decomposition characteristics of 9.5% C_4_F_7_N-9.5% O_2_-81% CO_2_ gas mixtures under arc discharge conditions and found that the decomposition of C_4_F_7_N mainly incorporated CO, CF_4_, C_2_F_4_, CF_3_CN, C_2_F_5_CN and C_2_N_2_ [4]. Furthermore, the particles produced by the decomposition of C_4_F_7_N under arc discharge conditions were not recoverable. Q.D. Huang et al. studied the overheated decomposition properties of a C_4_F_7_N/CO_2_/O_2_ gas mixture and found that its main decomposition product also contained C_2_N_2_ gas [5]. These results suggest that C_2_N_2_ can be used as a characteristic component of C_4_F_7_N decomposition that reacts to the aging state of C_4_F_7_N insulation under electrical and thermal faults.

C_2_N_2_ is highly toxic and readily reduced to cyanide. A. Beroual and C. Preve assessed the occupational exposure limit of 10 μL/L (8 h) with an inhalation test in rats and observed that the prolonged inhalation of C_2_N_2_ caused a break in the mitochondrial transfer chain of the rat cells and caused respiratory and visual irritation [6,7]. Meanwhile, it revealed that a break in the mitochondrial transfer chain of the rat cells and a strong irritation of the respiratory and visual systems, which could lead to health hazards and even death, was caused by the prolonged inhalation of C_2_N_2_. Therefore, it is particularly important to ensure the safety of the operation and the maintenance of the electrical equipment using C_4_F_7_N as the insulating medium.

MoS_2_, as a new semiconductor gas-sensitive material with a high surface area ratio and a natural semiconductor band gap [8], is broadly used in semiconductor-based micro gas-sensitive sensors as well as in the fabrication of adsorbents. However, the gas sensitivity of intrinsic MoS_2_ for specific gases is often unsatisfactory, and transition metal catalyst doping is used for gas sensitivity [9,10]. Cuprum (Cu) is a relatively inexpensive transition metal catalyst compared to precious metal catalysts such as aurum (Au), argentum (Ag) and platinum (Pt). Existing studies on the gas sensitivity of Cu and its cognate elements doped with MoS_2_ for specific gases demonstrate that Cu can be an excellent candidate for the MoS_2_ doping of two-dimensional materials. For example, the enhancement of the gas sensitivity of C_2_H_2_ by Au- and Ag-doped MoS_2_ was studied, and the results indicated that the doping of Au and Ag in the same main group can improve the gas sensitivity of dissolved gas C_2_H_2_ in insulating oil [11,12]. Furthermore, the gas sensitivity of pure and Cu-doped MoS_2_ to CO and NO was discussed in document [13]. It was found that Cu doped with sulfur (S) site can significantly improve the adsorption energy for CO and NO. Furthermore, according to the Hund’s rule of external electron configuration and the external electron configuration law, the external electron configuration formula of Cu is [Ar] 3d104s1. As far as transition metal elements are concerned, the outermost electron number of Cu is 1, which will make Cu atoms easily bond with the lone pair electrons of C, N and F atoms in the decomposition products of C_4_F_7_N, forming a strong adsorption force and achieving the purpose of capturing specific gases by gas-sensitive materials. It can be concluded that Cu may be the most ideal doping element to improve the gas sensitivity of MoS_2_ to C_4_F_7_N and its decomposition products.

The manuscript is structured as follows: First, the DMol^3^ module in Materials Studio software is used to model and calculate the stable structure of Cu cluster-modified MoS_2_. Second, the electronic density of states and the charge transfer amounts before and after the adsorption of C_4_F_7_N and C_2_N_2_ are compared to obtain the gas-sensitive mechanism of Cu-modified MoS_2_ for the detection of C_4_F_7_N and its decomposition products. Third, the gas-sensitive performance of the optimal doping system under different electric fields is investigated considering the complex working conditions of power equipment. This paper investigates the adsorption mechanism of the environmentally friendly insulating gas C_4_F_7_N and its decomposition product C_2_N_2_ on the Cu cluster-modified MoS_2_ surface through theoretical calculations and lays the groundwork for the gas content monitoring of the new electrical insulation equipment using C_4_F_7_N_2_.

**Figure 1:**
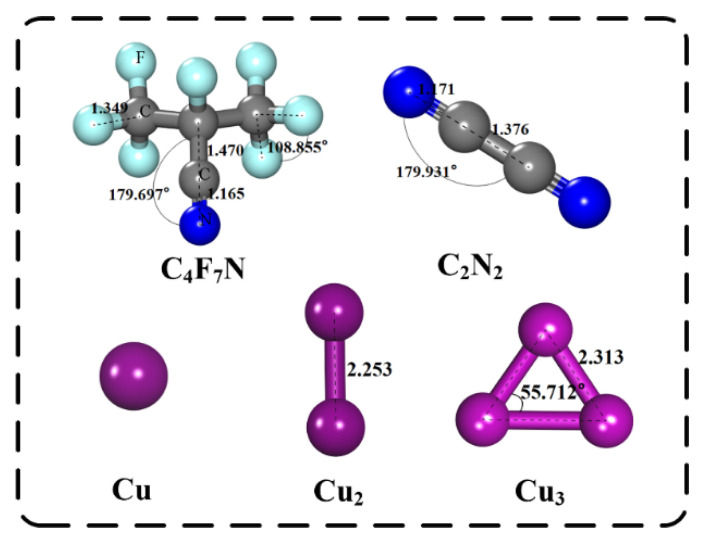
Structurally optimized molecular structures of C_4_F_7_N, C_2_N_2_ and Cu clusters. The distance is in Å.

The structure and energy optimization of the computational model was carried out based on the first principles of Density Functional Theory (DFT). We first constructed a 4 × 4 × 1 MoS_2_ supercell structure. Since it is necessary to find a section for quantum mechanical adsorption calculation, the (0,0,1) section was selected in this work, and a vacuum layer with a thickness of 15 Å was established to prevent reactions between different periods. Then, the PBE function based on DFT and the GGA of the spin polarization measurement were selected to deal with the calculation problem of electron crosslinking [14]. The molecular structures of C_4_F_7_N, C_2_N_2_ and Cu clusters were optimized, and the optimal structures are shown in Figure 1. Detailed bond lengths and bond angles of C_4_F_7_N, C_2_N_2_ and Cu cluster molecules are shown in Table 1.

The DFT-D2 method was used for dispersion correction [15], and the energy threshold, convergence force and self-consistent field were set as 1 × 10^−5^ Ha (1 Ha = 27.2114 eV), 2 × 10^−3^ Ha/Å and 1 × 10^−6^ eV, respectively, with a maximum displacement of 0.005 Å [16]. Since the formation of molecular orbits deforms atomic orbits to some extent, resulting in the deviation between the calculated results and the actual situation, double numerical polarization (DNP) was adopted to calculate the exchange electron pseudopotential to obtain more accurate results [17]. In orbital electronic processing, a single effective potential (DFT semicore Pseudopots (DSPP)) was used to replace the core electron to reduce the computational cost [18]. In the model surface calculation, the k point in the Brillouin region was set as 5 × 5 × 1 [13]. 

The stability of doped structures can be measured by binding energy (*E_b_*) [14], and it can be determined by Equation (1), as follows:

(1) $$E_{b}=E_{\left(\mathrm{Cu}\right)\gamma -\mathrm{MoS}2}-(E_{\mathrm{MoS}2}+E_{\left(\mathrm{Cu}\right)}{}_{\gamma })$$

where *E*_(Cu)*γ*-MoS2_, *E*_MoS2_ and *E*_(Cu)*γ*_ represent the binding energies of (Cu)_γ_-MoS_2_, pure MoS_2_ and the Cu cluster, respectively.

The adsorption energy (*E*_ads_) can be calculated by Equation (2), which is as follows:

(2) $$E_{\mathrm{ads}}=E_{(\mathrm{substrate}+\mathrm{gas}}-E_{\mathrm{substrate}}-E_{\mathrm{gas}})$$

where *E*_substrate+gas_ represents the total energy of the gas molecule adsorbed on the Cu_γ_-MoS_2_ surface, *E*_substrate_ and *E*_gas_ are the energies of Cu_γ_-MoS_2_ and a gas molecule, respectively.

The charge amount is obtained by Mullikan [12] charge analysis. The transferred charge (*Q_t_*) is determined by Equation (3), which is as follows:

(3) $$Q_{t}=Q_{\mathrm{ads}}-Q_{\mathrm{iso}}$$

where *Q*_ads_ and *Q*_iso_ represent the total charge of gases after and before adsorption, respectively.

## 2. Results and Discussion

The adsorption energy *E*_ads_ proves whether the reaction can be spontaneous and indicates the strength of the adsorption reaction [19]. If *E*_ads_ < 0, the reaction releases energy, indicating that the adsorption reaction can be spontaneous. The transferred charge quantity *Q_t_* can be used to explain the charge exchange level between the gas molecule and the substrate and then to explain the change in the conductivity of the system. 

### 2.1. Construction of Stable Structures of Cu Clusters-Doped MoS_2_

**Figure 2:**
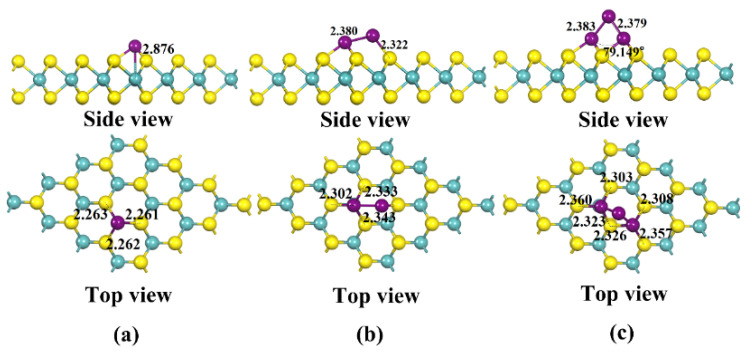
Three stable structures of MoS_2_ modified by Cu clusters: (**a**) Stable structure of Cu-modified MoS_2_; (**b**) Stable structure of Cu_2_-modified MoS_2_; (**c**) Stable structure of Cu_3_-modified MoS_2_.

**Figure 3:**
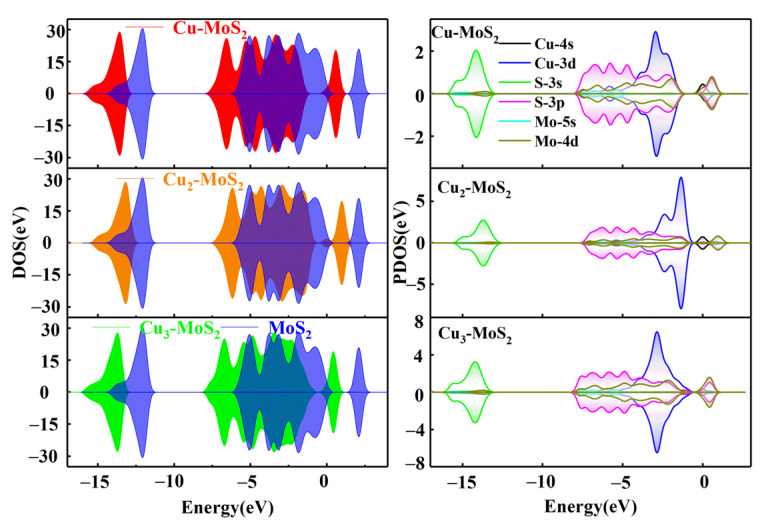
Electron density of states and partial density of states before and after the modification of MoS_2_ by Cu clusters.

The most stable modified structure obtained by calculating the binding energy of Cu clusters doped at different positions, such as Sulfur (S), Molybdenum (Mo) and bridge positions according to Equation (1), is shown in Figure 2. The yellow atom in the diagram is S, and the cyan atom is Mo. The binding energies of the Cu clusters with MoS_2_ are calculated from Equation (1) to be −1.723, −1.831 and −2.976 eV, respectively, all of which are greater than −0.6 eV [19], indicating that the structure has strong stability. Three S-Cu bonds with bond lengths of 2.261, 2.263 and 2.262 Å and a Mo-Cu bond of 2.876 Å are formed on the MoS_2_ surface by single Cu atoms. Meanwhile, four S-Cu bonds with lengths of 2.302, 2.333, 2.343 and 2.322 Å are formed between Cu_2_ and the S atoms of MoS_2_. Interestingly, the bond length of Cu_2_ increased significantly from 2.253 Å before modification to 2.380 Å, indicating that Cu-Cu is activated, which is strong evidence of its stability in binding to MoS_2_. Similarly, Cu_3_ breaks a Cu-Cu bond upon binding to MoS_2_, forming six S-Cu bonds with bond lengths of 2.303, 2.360, 2.323, 2.326, 2.308 and 2.357 Å, respectively. Further, the electronic density of states (DOS) and partial density of states (PDOS) distributions for (Cu)_γ_-MoS_2_ and pure MoS_2_ are obtained, which are shown in Figure 3.

As shown in Figure 3, the electron density distribution of MoS_2_ changes significantly after the Cu_γ_ (γ = 1, 2, 3) modification, which is reflected in the significant leftward shift of the DOS diagram compared to the intrinsic MoS_2_, resulting in a decrease in the electron energy level. Based on the PDOS diagram of Cu-MoS_2_ in Figure 3, it can be seen that the electron densities of Cu-3d and S-3p peak simultaneously and are close to −6, −4 and −2 eV, indicating that strong orbital hybridization between the two has been formed. The two orbit peaks of S-3p and Mo-4d at 2 eV overlap. The aforementioned facts reasonably explain the bond forming between the Cu and S atoms in Cu-MoS_2_. 

As far as the Cu_2_-MoS_2_ system is concerned, the electron density increases near the Fermi level (*E* = 0 eV), which indicates that there is an increase in conductivity for the Cu_2_-modified system. In terms of electron orbit analysis, the Cu-3d, Mo-4d and S-3p orbits have strong orbital hybridization around −1.5 eV. The electron density contribution from the Cu-4s orbit is responsible for the elevated electron density near the Fermi level. The PDOS diagram of the Cu_3_-MoS_2_ system shows that the Cu-3d, Mo-4d and S-3p orbits reach a simultaneous maximum in electron density at −3.5 eV, which accounts for the bonding of Cu to S atoms.

### 2.2. Adsorption on the Cu-MoS_2_ Monolayer

**Figure 4:**
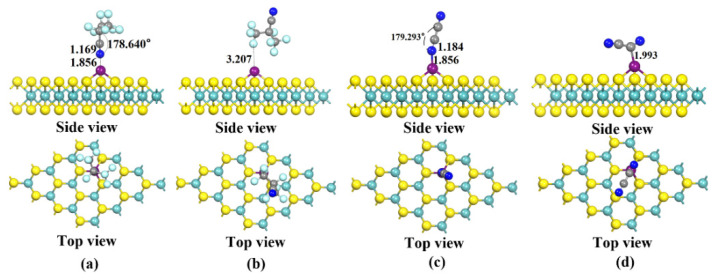
Four stable structures of Cu-modified MoS_2_ for C_4_F_7_N and C_2_N_2_ adsorption obtained after structural optimization: (**a**) X11: Stable structure of C_4_F_7_N adsorbed on Cu-MoS_2_ surface; (**b**) X12: Stable structure of C_4_F_7_N adsorbed on Cu-MoS_2_ surface; (**c**) X21: Stable structure of C_2_N_2_ adsorbed on Cu-MoS_2_ surface; (**d**) X22: Stable structure of C_2_N_2_ adsorbed on Cu-MoS_2_ surface.

**Figure 5:**
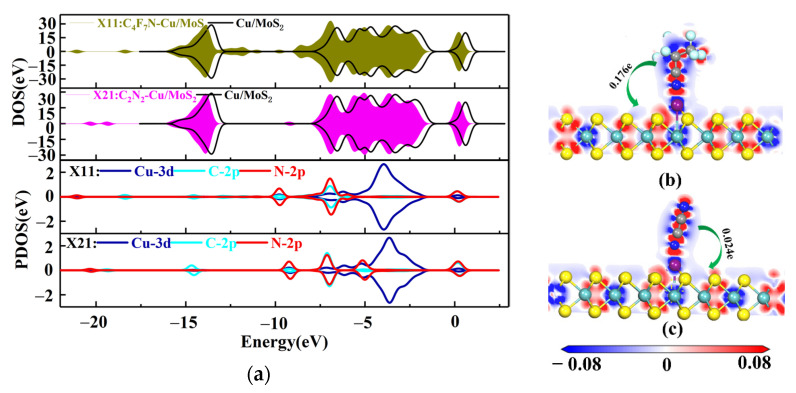
(**a**) DOS and PDOS plots of X11 and X21; (**b**) EDD plots for X11 (**c**) EDD plots for X21.

Figure 4 shows the four different conformations of C_4_F_7_N and C_2_N_2_ adsorbed on the Cu-MoS_2_ surface, and the candidates are labeled as X11, X12, X21 and X22, respectively. The magnitudes of the adsorption energies of the four systems calculated with Equation (2) are as follows: *E*_ads_(X21) = −1.080 eV > *E*_ads_(X11) = −1.011 eV > *E*_ads_(X22) = −0.632 eV > *E*_ads_(X12) = −0.235 eV. According to the calculated adsorption energy, the adsorption strength of the Cu-MoS_2_ system is slightly greater than that of C_4_F_7_N for C_2_N_2_, and both of them are spontaneous adsorption. Here, only X11 and X21, which have larger adsorption energies, are taken as examples for further analysis. The minimum adsorption distance between C_4_F_7_N and C_2_N_2_ on the Cu-MoS_2_ surface is 1.856 Å, and the electron density distribution after adsorption is shifted to the left, as shown in Figure 4 and Figure 5a. The electron difference density (EDD) plots for X11 and X21 are shown in Figure 5b,c, where the red region indicates electron loss and the blue region indicates electron enrichment. The C and N atoms of CN* of C_4_F_7_N in X11 are surrounded by the red region, indicating electron loss, and the Cu atoms are surrounded by the blue electron cloud, indicating electron gain by the Cu atom. In contrast, the red region in X21 is distributed around the two CN* of C_2_N_2_, which demonstrates an overall loss of electrons and a transfer of electrons to the substrate.

For X11, the C-2p and N-2p orbits near −9.5 and −4 eV have a clear overlap of orbit peaks with the Cu-3d orbit, forming a strong orbital hybridization, as shown by the DOS and PDOS distributions in Figure 5a. The orbital overlap of Cu-3d with N-2p near the Fermi energy level reasonably explains the bonding of the N atom of CN* to the Cu atom in C_4_F_7_N. Based on the amount of transferred charge charge of X11 (0.176e), C_4_F_7_N is positively charged after adsorption, indicating that it acts as an electron donor transferring electrons to the substrate, which results in an increase in the conductivity of the substrate. Similarly, for the X21 system, the orbital hybridization of C-2p, N-2p and Cu-3d is mainly at −8 and −4.5 eV. Unlike C_4_F_7_N, the orbital hybridization of N-2p and Cu-3d after the adsorption of C_2_N_2_ on the Cu-MoS_2_ surface is mainly concentrated near the −5 eV, which is closer to the Fermi energy level, and this is the reason why the surface interaction between C_2_N_2_ and Cu-MoS_2_ is stronger than that of C_4_F_7_N. The transferred charge of C_2_N_2_ is 0.024e, again acting as an electron donor to the substrate.

### 2.3. Adsorption on the Cu_2_-MoS_2_ Monolayer

**Figure 6:**
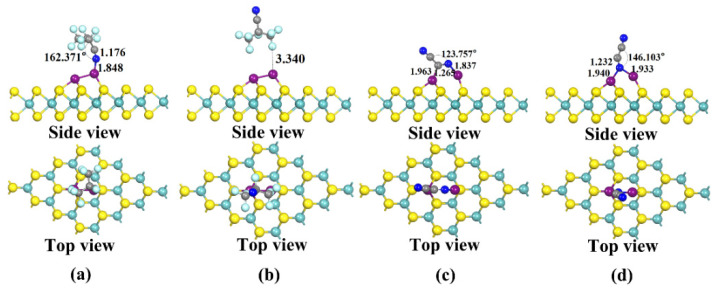
Four stable structures of Cu_2_-modified MoS_2_ for C_4_F_7_N and C_2_N_2_ adsorption obtained after structural optimization: (**a**) X31: Stable structure of C_4_F_7_N adsorbed on Cu_2_-MoS_2_ surface; (**b**) X32: Stable structure of C_4_F_7_N adsorbed on Cu_2_-MoS_2_ surface; (**c**) X41: Stable structure of C_2_N_2_ adsorbed on Cu_2_-MoS_2_ surface; (**d**) X42: Stable structure of C_2_N_2_ adsorbed on Cu_2_-MoS_2_ surface.

**Figure 7:**
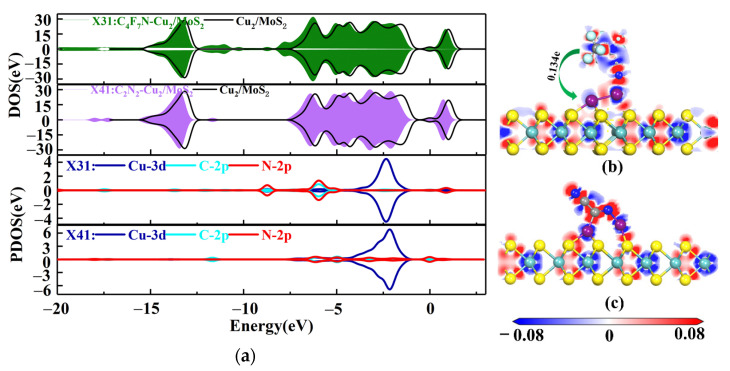
(**a**) DOS and PDOS plots of X31 and X41; (**b**) EDD plots for X31; (**c**) EDD plots for X41.

The four stable structures of C_4_F_7_N and C_2_N_2_ adsorbed on the Cu_2_-MoS_2_ surface are shown in Figure 6. They are named as X31, X32, X41 and X42, respectively. According to Equation (2), the calculated magnitudes of the adsorption energies are as follows: *E*_ads_(X41) = −1.572 eV > *E*_ads_(X31) = −0.956 eV > *E*_ads_(X42) = −0.6–0.662 eV > *E*_ads_(X32) = −0.184 eV. Here, only X41 and X31, which have a higher adsorption energy, are taken for further analysis. Figure 7 shows the DOS and PDOS of X41 and X31 after adsorption. Based on the DOS diagram shown in Figure 7a, the variation in the density of electron states from −2 to −4 eV after the adsorption of C_2_N_2_ is more pronounced than that of C_4_F_7_N, which reasonably indicates that the adsorption of C_2_N_2_ with the Cu_2_-MoS_2_ surface is stronger than that of C_4_F_7_N. In detail, with the PDOS diagram shown in Figure 7, the X31 structure has orbital hybridization present at −8 and −6 eV for C-2p, N-2p and Cu-3d. The X41 system has orbital hybridization at −6 and −5 eV for C-2p, N-2p and Cu-3d, and it is close to the Fermi energy level. The transferred charge of C_2_N_2_ in the X41 system is −0.186e after adsorption by the Cu_2_-MoS_2_ surface, which acts as an electron acceptor, and electrons are transferred from the substrate to the C_2_N_2_ molecule, resulting in a decrease in the substrate conductivity. The transferred charge (0.134e) and adsorption energy of the system of X31 are both reduced compared to the system of X11. The EDD plots for X31 and X41 are shown in Figure 7b,c. The N atom of the CN* of C_4_F_7_N in X31 is surrounded by a red region, indicating electron loss, while the Cu atom is surrounded by a blue electron cloud, indicating electron gain from the Cu atom. The red region in X41 is distributed around the CN* of C_2_N_2_, which proves its loss of electrons and the transfer of electrons to the two Cu atoms.

### 2.4. Adsorption on the Cu_3_-MoS_2_ Monolayer

**Figure 8:**
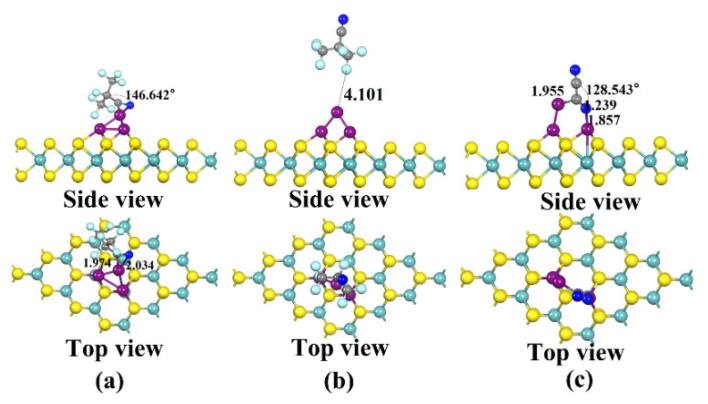
Three stable structures of Cu_3_-modified MoS_2_ for C_4_F_7_N and C_2_N_2_ adsorption obtained after structural optimization: (**a**) X51: Stable structure of C_4_F_7_N adsorbed on Cu_2_-MoS_2_ surface; (**b**) X52: Stable structure of C_4_F_7_N adsorbed on Cu_3_-MoS_2_ surface; (**c**) X61: Stable structure of C_2_N_2_ adsorbed on Cu_3_-MoS_2_ surface.

**Figure 9:**
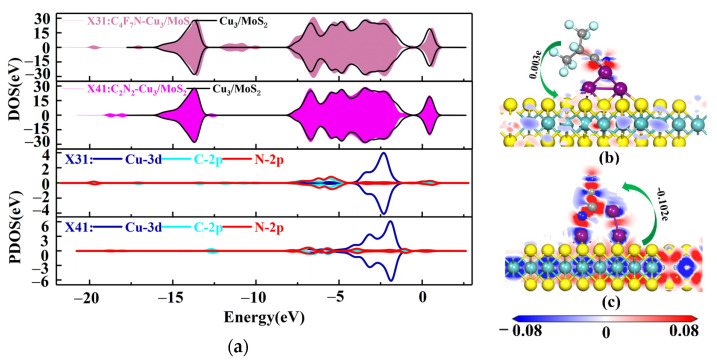
(**a**) DOS and PDOS plots of X51 and X61; (**b**) EDD plots for X51; (**c**) EDD plots for X61.

The three stable structures of the two gases adsorbed on the Cu_3_-MoS_2_ surface, C_4_F_7_N and C_2_N_2_, are shown in Figure 8 and are named X51, X52 and X61, respectively. The adsorption energies are listed as follows: *E*_ads_(X61) = −1.09 eV > *E*_ads_(X51) = −0.770 eV > *E*_ads_(X52) = −0.239 eV. For X51, the C_4_F_7_N gas molecule undergoes a large change in shape after adsorption. This is demonstrated by the reduction in the bond angle of C-C≡N from 179.697° to 146.642° before adsorption and the shortest distance for adsorption of 1.974 Å. The PDOS diagram, shown in Figure 9a, shows that the C-2p, N-2p and Cu-3d orbital hybridization is mainly concentrated in the −4 to −7.5 eV range. From the calculated adsorption energy magnitude, it is learned that the adsorption energy of the X51 system is less than −0.6 eV, which means that chemisorption can no longer be constituted. In contrast, the degree of change in the structure of X61 after C_2_N_2_ adsorption is more pronounced compared to that of X51. In detail, the bond angle of C_2_N_2_ of C-C≡N drops sharply from 179.931° before adsorption to 128.543° and forms a “ring” structure with the Cu_3_ cluster. At the same time, orbital hybridization exists at −5.5 to −7 eV and at −3.5 eV for C-2p, N-2p and Cu-3d. According to the EDD diagram shown in Figure 9b,c, it can be seen that the distribution of the electron cloud around the C_4_F_7_N molecule in X51 is not obvious, indicating a relatively small amount of charge transfer. The blue region in X61 is distributed around C in the CN* above C_2_N_2_, which proves that it has gained electrons.

**Figure 10:**
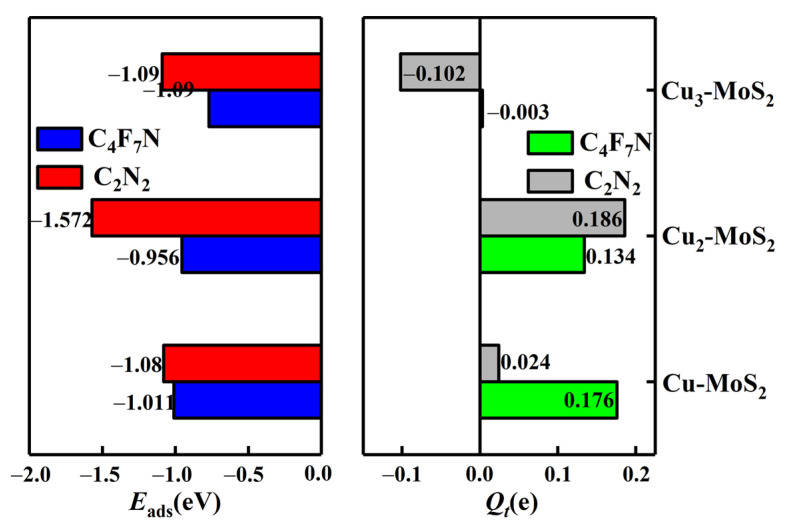
Maximum adsorption energies of C_4_F_7_N and C_2_N_2_ on Cu_γ_ (γ = 1–3)-MoS_2_ surfaces and charge transfer in the corresponding systems.

From Figure 10, it can be seen that the maximum adsorption energy of Cu_γ_-MoS_2_ for C_4_F_7_N shows a decreasing trend as the value of the Cu cluster *γ* (*γ* = 1, 2, 3) increases. The transferred charge is 0.003e, tending to 0, which has less of an effect on the conductivity of the substrate. At this point, Cu_γ_-MoS_2_ can still satisfy the chemisorption for C_2_N_2_ with good selectivity. Therefore, Cu_3_ small clusters modified with MoS_2_ can avoid main gas interference and be used as a sensitive substrate for the detection of C_2_N_2_-resistive gas sensors.

### 2.5. Externally Applied Electric Field

**Figure 11:**
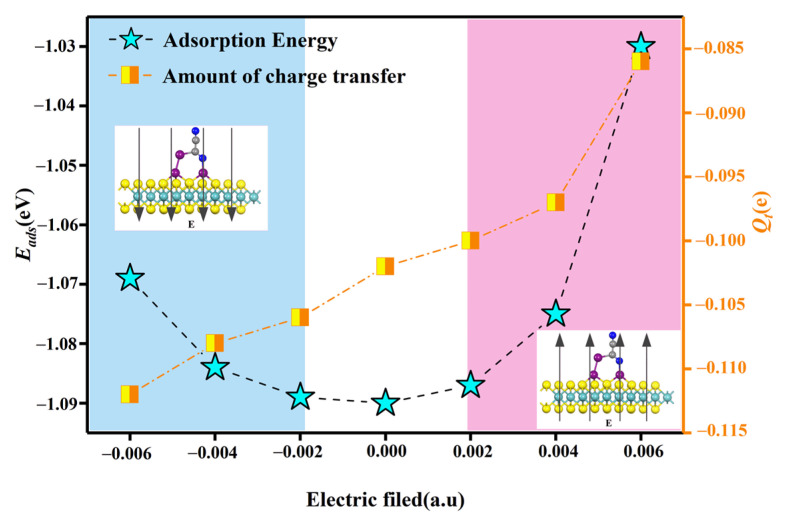
The effect of an applied electric field on the adsorption energy and the amount of charge transfer in the X61 system.

This is investigated by applying positive and negative polar electric fields perpendicular to the X61 system. The effect of the externally applied electric field on the adsorption energy and charge transfer of X61 is shown in Figure 11. In Figure 11, 1 a.u is equal to 51.36 V/Å. As can be seen from Figure 11, the application of either a positive or negative electric field is detrimental to the adsorption energy, resulting in a reduction in the adsorption energy. However, when a positive polar electric field is applied, the electrons due to the electric field force cause the electrons gained by the C_2_N_2_ gas molecules to increase with the increase in the electric field value. In general, although the adsorption energy and the magnitude of the transferred charge are influenced by the applied electric field, they still meet the detection requirements.

### 2.6. Band Strcture and Recovery Time of C_2_N_2_-Cu_3_/MoS_2_

**Figure 12:**
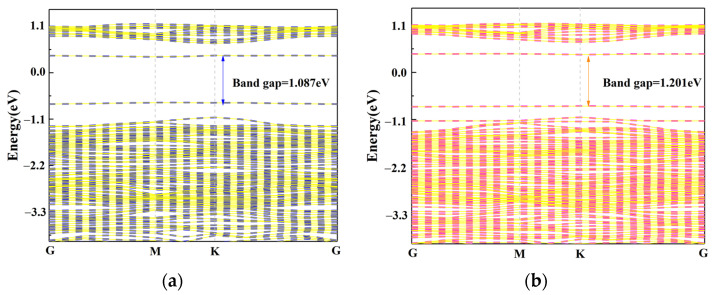
Band structure: (**a**) Band structure of Cu_3_/MoS_2_; (**b**) Band structure of C_2_N_2_-Cu_3_/MoS_2_.

We calculated the change in the band gap values before and after the adsorption of C_2_N_2_ gas on the Cu_3_/MoS_2_ surface are shown in Figure 12. In this case, the Brillouin zone scan path was set to G-M-K-G during the calculation of the bandgap map. The calculated band gap value of the adsorbed C_2_N_2_ gas molecules is 1.201 eV, which is 10.5% higher than the band gap value of 1.087 eV of the Cu_3_-MoS_2_ system before adsorption, which is consistent with our previous finding that C_2_N_2_ acts as an electron acceptor and thus leads to a decrease in the conductivity of the Cu_3_-MoS_2_ system. Meanwhile, we calculated from Equation (4) that the desorption time of C_2_N_2_ at 370 K is 645 s, which can have good recovery characteristics.

The general formula for calculating the recovery time of a gas-sensitive substrate is defined as [20,21]:

(4) $$\tau =\frac{e^{-E_{\mathrm{ads}}/KT}}{v_{0}}$$

where *υ*_0_ is the apparent frequency (here, we take 10^12^ s^−1^); *E*_ads_ is the adsorption energy; *K* is the Boltzmann constant (8.62 × 10^−5^ eV/K); *T* is the temperature (K).

## 3. Synthesis of Cu Clusters-Doped MoS_2_ Gas-Sensitive Materials

**Figure 13:**
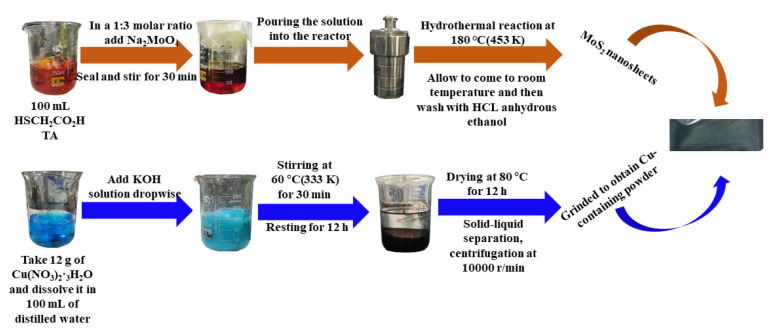
Flow chart of the Cu-containing cluster-doped MoS_2_ synthesis.

In this paper, Cu-containing cluster-doped MoS_2_ gas-sensitive materials were synthesized at the experimental level. The specific synthesis process is shown in Figure 13. The analytical pure grade drugs involved in the synthesis of this paper were purchased from Shanghai Aladdin. For the synthesis of MoS_2_ nanosheets, 100 mL of thioglycolic acid (HSCH_2_CO_2_H, TA) was first placed in a 250 mL volumetric beaker, and sodium molybdate (Na_2_MoO_4_) was added to the above beaker in a 1:3 molar ratio with TA, sealed and stirred for 30 min at room temperature using a magnetic stirrer. For the preparation of the Cu-containing powder, 12 g of Cu(NO_3_)_2_·3H_2_O was first dissolved in 100 mL of distilled water and stirred for 15 min before adding potassium hydroxide (KOH) solution dropwise; the solution gradually became darker in color, and after warming and stirring at 60 °C (333 K), the solution turned black and was left to stand for 12 h to obtain a solid-liquid layered solution. The upper layer of the solution was removed with a dropper, and the solid was centrifuged in a centrifuge at 10,000 r/min, put into an oven, dried at 80 °C for 12 h and ground to obtain Cu powder.

**Figure 14:**
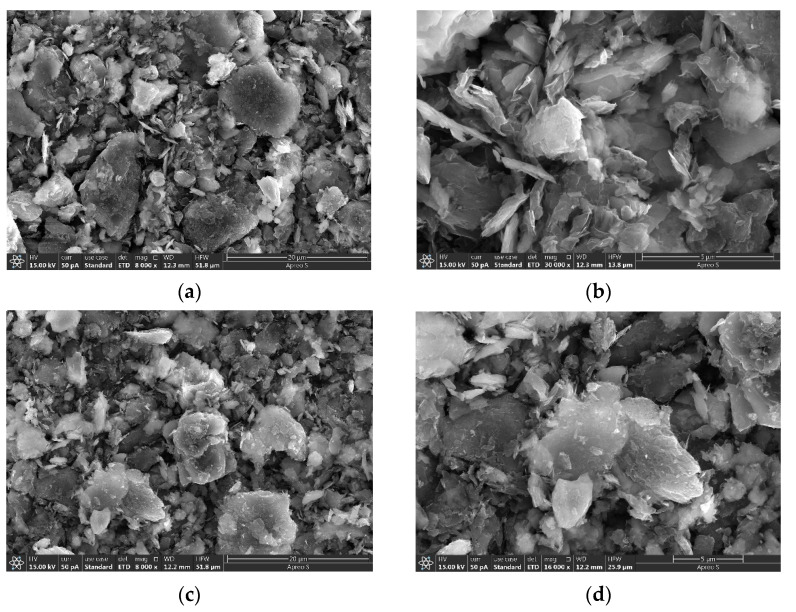
Microscopic patterns of MoS_2_ nanosheets and Cu-containing cluster-doped MoS_2_: (**a**) Microscopic representation of MoS_2_ nanosheets at a 20 µm scale; (**b**) Microscopic representation of MoS_2_ nanosheets at a 5 µm scale; (**c**) Cu-containing cluster-doped MoS_2_ at a 20 µm scale; (**d**) Cu-containing cluster-doped MoS_2_ at a 5 µm scale.

For the synthesis of Cu-containing cluster-doped MoS_2_, the gas-sensitive material was mainly obtained by mixing the abovementioned synthesized Cu-containing powder as well as MoS_2_ nanosheets in a 1:9 molar ratio into isopropanol (C_3_H_8_O), stirring for 8 h using a magnetic stirrer and then drying. The synthesized MoS_2_ and the microscopic appearance of the Cu-containing cluster-doped MoS_2_ are shown in Figure 14. The scanning electron microscope (SEM) image in Figure 14 shows that the MoS_2_ exhibits a sheet-like structure with a smooth surface. In contrast, the doped MoS_2_ nanosheets have many tiny clusters attached to them, resulting in a Cu-containing cluster-doped MoS_2_ composite structure.

## 4. Conclusions

In this paper, the adsorption mechanism of C_4_F_7_N and its electrothermal aging decomposition characteristic component C_2_N_2_ on Cu_γ_ (γ = 1–3)-MoS_2_ are investigated based on the DFT theory by calculating the DOS, the PDOS and the adsorption energy. The following conclusions are obtained.

The maximum adsorption energy of a single Cu atom modified with MoS_2_ is −1.011 and −1.080 eV for C_4_F_7_N and C_2_N_2_, respectively. Due to the close proximity of the two values, the selectivity requirements for detection may not be met at this point. However, as the number of doped copper atoms increases, the adsorption energy of the doped system for C_4_F_7_N gradually decreases, reaching a minimum value of −0.770 eV at *γ* = 3. In comparison, the magnitude of the adsorption energy of C_2_N_2_ is significantly higher than that of C_4_F_7_N at −1.09 eV. The calculated band gap value of the C_2_N_2_ gas molecules after adsorption increased by 10.5% compared to the Cu_3_-MoS_2_ system before adsorption. This indicates that a more significant decrease in conductivity occurred after adsorption. Moreover, the desorption time of C_2_N_2_ at 370 K is 645 s, which can have good recovery characteristics. Small clusters of Cu_3_-modified MoS_2_ are therefore suitable as detection gas-sensitive substrates for the detection of the toxic gas C_2_N_2_.

When an electric field of positive and negative polarity is applied to Cu_3_-MoS_2_, the adsorption energy of C_2_N_2_ on the surface of Cu_3_-MoS_2_ decreases with the increase in the electric field, but it is still greater than −0.6 eV, which still meets the detection requirements. When a negative polarity electric field is applied, *Q_t_* increases as the electric field increases due to the force of the electric field. The conclusions of this paper provide theoretical guidance for the selection of gas-sensitive materials for the decomposition products of C_4_F_7_N.

## Figures and Tables

**Table 1:** Bond lengths and bond angles of C_4_F_7_N, C_2_N_2_ and Cu cluster molecules.

Molecular	Bond Length (Å)	Bond Angle (°)
C_4_F_7_N	d _(C≡N)_ = 1.165 d _(C-F)_ = 1.349 d _(C-C)_ = 1.470	∠ _(C-C-N)_ = 179.697 ∠ _(F-C-F)_ = 108.855
C_2_N_2_	d _(C-C)_ = 1.376 d _(C≡N)_ = 1.171	∠ _(C-C-N)_ = 179.831
Cu_2_	d _(Cu-Cu)_ = 2.253	-
Cu_3_	d_(Cu-Cu)_ = 2.313	∠ _(Cu-Cu-Cu)_ = 55.712

## Data Availability

The data that support the findings of this study are available from the corresponding author, [C.L. (Changyun Li)], upon reasonable request.
